# Selisistat, a SIRT1 inhibitor, enhances paclitaxel activity in luminal and triple-negative breast cancer: in silico, in vitro, and in vivo studies

**DOI:** 10.1080/14756366.2025.2458554

**Published:** 2025-02-12

**Authors:** Anna Wawruszak, Jarogniew Luszczki, Damian Bartuzi, Joanna Kalafut, Estera Okon, Arkadiusz Czerwonka, Andrzej Stepulak

**Affiliations:** ^a^ Department of Biochemistry and Molecular Biology, Medical University of Lublin, Lublin, Poland; ^b^ Department of Occupational Medicine, Medical University of Lublin, Lublin, Poland; ^c^ Department of Synthesis and Chemical Technology of Pharmaceutical Substances with Computer Modelling Laboratory, Medical University of Lublin, Lublin, Poland; ^d^ Science for Life Laboratory, Department of Cell and Molecular Biology, Uppsala University, Uppsala, Sweden

**Keywords:** Breast cancer, paclitaxel, selisistat, sirtuin inhibitor, histone deacetylase inhibitor

## Abstract

Sirtuins (SIRTs) are NAD+-dependent histone deacetylases, which play a key role in cancer progression; however, their prognostic values in breast cancer (BC) remain a subject of debate and controversy. Accumulative evidence suggests that each sirtuin possesses individual character, implicating its role in the regulation of multifaceted biological functions leading to BC initiation, progression and metastasis. Selisistat (EX527) is a potent, cell permeable, highly selective SIRT1 inhibitor. In the study, the tumour-suppressive effects of the SIRT1 inhibitor EX527 (selisistat) alone and in combination with paclitaxel (PAX) in different breast cancer cell lines and zebrafish xenograft models were investigated. The type of pharmacological drug-drug interaction between EX527 and PAX was determined using the isobolographic method. EX527 and PAX used individually inhibited proliferation, induced apoptosis and caused cell cycle arrest in G1 and subG1/G2 phases. Interestingly, the combination of these compounds used in the 1:1 dose-ratio augmented all these effects (IC_50add_ 29.52 ± 3.29 − 38.45 ± 5.26). The co-treatment of EX527 with PAX generated desirable additive drug-drug interaction. The simultaneous application of EX527 and PAX induced a stronger inhibition of tumour growth compared to individual treatments in zebrafish xenografts. *In silico* analysis revealed a protein-protein interaction pathway (SIRT1-AKT-S1PR1-GNAI1/GNAO1-Tubulin) connecting molecular targets of both ligands. To summarise, the combination of EX527 and PAX more effectively impairs breast cancer cell growth compared to individual treatments. However, further investigations are required to clarify the specific targets and molecular mechanisms underlying the activity of EX527:PAX in other preclinical models.

## Introduction

Breast cancer (BC) is the most commonly diagnosed type of neoplasm and the leading cause of mortality among women globally^1^. Unfortunately, about 10–15% of women will develop this disease in their lifetime^2^. Therefore, further efforts are necessary to counteract its growing burden, particularly in developed countries where the incidence of BC is increasing rapidly, and mortality rates remain at a very high level^1^. Significant progress in the understanding molecular mechanism of cancer growth and clinical management of BC has occurred over the past two decades, driving substantial developments in early detection and personalised BC therapy. However, the primary impediment in clinical practice is the intra- and intertumoral heterogeneity of BC^3^. BC comprises a group of diseases with specific molecular, histopathologic and clinical properties^4^. By using hierarchical clustering analysis of gene expression profiling, molecular subclasses (e.g. luminal, HER2-enriched, basal-like and normal-like) of BC were defined, each with distinctive biological and clinical features. This molecular classification has demonstrated prognostic value and predictive ability for chemotherapy response in the clinical setting^5^^,^^6^. Despite significant advances in the treatment of luminal and HER2-enriched BC, triple-negative breast cancer (TNBC) therapy primarily focuses on standard chemotherapy, which is associated with serious adverse effects. On the other hand, patients with luminal or HER2-enriched subclasses develop drug resistance over time, rendering further therapy non-effective. To overcome drug resistance and reduce toxic doses of standard therapeutics, chemotherapeutic drugs are being combined with other natural or synthetic medications. Currently, novel targeted therapies with PAX are being tested to achieve better clinical outcomes in patients with BC^7^.

**Figure 1.**
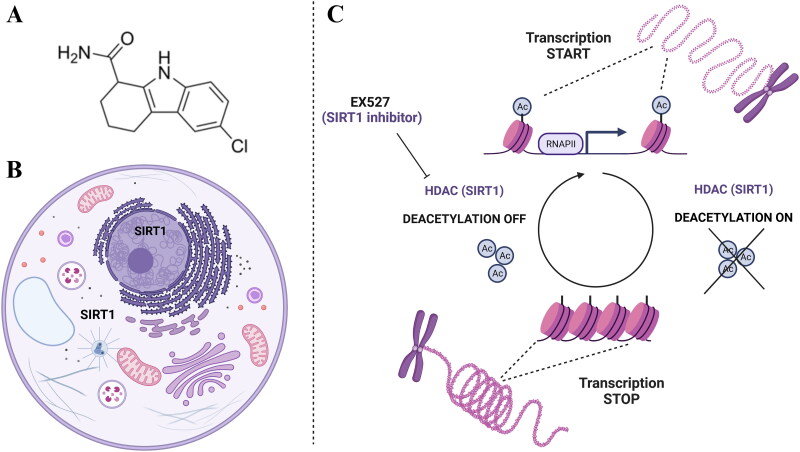
Histone acetylation modifications in cancer biology. (a) The chemical structure of selisistat (EX527) – sirtuin 1 (SIRT1) inhibitor. (b) Cellular localisation of SIRT1. (c) Inhibition of histone deacetylation (DEACETYLATION OFF) (catalyzed by SIRT1 (HDAC)) through EX527. After inhibition, HDACs are unable to remove acetyl groups (Ac) from the N-terminus lysine residues. This leads to an opened state of the chromatin and increases transcriptional activity of RNA polymerase II (RNAPII).

Chromatin remodelling plays a crucial role in carcinogenesis. Recent findings indicate that epigenetic modifications are key factors in BC development. These alterations are particularly attractive as therapeutic targets due to their potential for reversal^8^. Histone tail modifications are precisely controlled by histone-modifying enzymes, and disruptions in their activity contribute to neoplastic processes. Acetylation of histones is mediated by histone acetyltransferases (HATs), which biological activity is counteracted by histone deacetylases (HDACs). In contrast to HATs, the activity of HDACs leads to a repressive chromatin state^9^. An imbalance between HATs and HDACs has been reported in the development and progression of BC (Figure 1)^7^.

Sirtuins, also known as silent information regulators (SIRTs), belong to the III class of HDACs and play a role in the epigenetic modifications of proteins contributing to multiple biological processes. SIRTs are involved in metabolic transformations as well as regulation of the cell cycle, proliferation, viability, or cell death. Among four classes of HDACs, only SIRTs require nicotinamide adenine dinucleotide (NAD+) as a co-factor to hydrolyse acylated amino acid residues. Interestingly, SIRTs are not only engaged in the deacetylation of histones, but also of other proteins, including transcription factors or enzymes. Molecular phylogenetic analysis categorises mammalian SIRTs into four classes: I (SIRT1, SIRT2 and SIRT3), II (SIRT4), III (SIRT5) and IV (SIRT6 and SIRT7)^10^^,^^11^. Inhibitors of SIRTs (SIRTi) have been actively pursued and intensively studied in recent years as a novel form of therapy for cancer or neurodegenerative diseases^12^^,^^13^. A variety of SIRT1 inhibitors, including sirtinol, cambinol, suramin, salermide, splitomicin, tenovin, indole derivatives, and structurally related analogues, have been designed and tested. Both *in vitro* and *in vivo* studies involving oestrogen receptor positive and oestrogen receptor negative cell lines have demonstrated that SIRT1 inhibition suppresses BC cell proliferation and induces apoptosis. This is often mediated through p53-dependent pathways, where SIRT1 inhibition increases acetylation of p53 at lysine 382 (p53K382), thereby enhancing its pro-apoptotic activity. In certain cases, apoptosis can also occur independently of p53, as SIRT1 inhibitors reactivate pro-apoptotic genes, such as the CASP genes encoding caspase-3, −8, and −9, which were previously epigenetically repressed by SIRT1^14^. The activity of SIRT1 inhibitors has also been demonstrated^15^ in combined therapy with other active agents^16–18^ in hepatocellular carcinoma^19^, oral squamous cell carcinoma^20^, oropharyngeal cancer^21^, oesophageal cancer^22^^,^^23^ or lung cancer^24^. These findings underscore the potential of SIRT1 inhibitors as antitumor agents in BC^14^.

Selisistat (EX527) is the first identified cell permeable selective SIRT1 inhibitor (Figure 1). Its therapeutic potential has been explored in numerous animal models for various pathologies, including cancers^25^^,^^26^. Studies have shown that EX527 induces apoptosis in glioma by activating the p53 signalling pathway^27^ or suppresses cell migration by inhibiting HSF1 protein stability in cervical cancer^28^. However, there is a lack of detailed studies on the effects of EX527 alone or in co-therapy with other drugs on BC cells.

Combinations of epigenetic targeted therapies with conventional chemotherapeutic drugs offer a future strategy to re-sensitize drug-resistant neoplasms, potentially act additively or synergistically to increase the therapeutic index^8^. In the study, the growth inhibition activity of EX527 when used individually or in combination with paclitaxel (PAX) under different cell culture conditions, including 2D *in vitro* and zebrafish xenograft *in vivo* models, were studied. The detailed pharmacodynamic drug-drug interactions between EX527 and PAX combinations were analysed by the advanced isobolographic method. Furthermore, putative favourable drug interactions between the molecular targets of EX527 and PAX were described.

## Materials and methods

### Pharmaceutical preparation

EX527 and PAX (Sigma; St. Louis, MO, USA) were dissolved in dimethyl sulfoxide (DMSO) (Sigma) to prepare 10 mM and 1 mM stock solutions, respectively. The studied substances were diluted in the recommended growth medium to different working concentrations before experiments, as indicated in the assays.

### Cell lines

The human T47D, MCF7 BC luminal A, MDA-MB-231, MDA-MB-468, BT-549 TNBC and BJ normal human skin fibroblast cell lines were obtained from the American Type Culture Collection (ATCC) (Manassas, VA, USA). The cells were cultured in the Dulbecco’s Modified Eagle Medium (DMEM)/Ham’s Nutrient Mixture F-12 medium (Sigma) supplemented with 10% foetal bovine serum (FBS) (Sigma), penicillin (100 units/ml) (Sigma), streptomycin (100 μg/ml) (Sigma) and incubated at 37 °C with 5% CO_2_.

### MTT assay

T47D, MCF7, MDA-MB-231, MDA-MB-468, BT-549 BC and BJ normal cells were seeded (1 × 10^4^ cells/ml) in 96-well plates (Nunc; Roskilde, Denmark) and kept in a standard growing state for 24 h. The next day, the culture media were changed, and the cells were treated with different concentrations of EX527 and PAX (individually or in combinations). Cell viability was determined after 96 h in the MTT (3–(4,5-dimethylthiazol-2-yl)-2,5-diphenyltetrazolium bromide) assay in which the yellow tetrazolium salt (MTT) was metabolised by viable cells to purple formazan crystals. 10 μl of MTT solution was added to each well and incubated at 37 °C for 4 h. Then, formazan crystals were solubilised overnight in a sodium dodecyl sulphate (SDS) buffer (10% SDS in 0.01 N HCl). Absorbance was measured at 570 nm by an Infinite M200 Pro microplate reader (Tecan Group Ltd.; Seestrasse, Switzerland). The dose-response curves (DRCs) created in the GraphPad Prism 5.0 program were used to determine median inhibitory concentration (IC_50_) values for EX527 and PAX.

### BrdU assay

The proliferation activity of BC cells after drug treatment was determined using the Cell Proliferation ELISA, 5-bromo-2-deoxyuridine (BrdU) Kit (Roche Diagnostics; Mannheim, Germany). The T47D, MCF7, MDA-MB-231, MDA-MB-468 and BT-549 BC cells were seeded at a density of 1 × 10^4^/ml on the 96-well plate (100 μl/well) and exposed to EX527 and PAX (1/2 IC_50_ and IC_50_) individually or combined for 48 h followed by incubation with 100 μM of BrdU. Then, the cells were fixed for 30 min. in the FixDenat solution. Monoclonal goat anti-mouse IgG anti-BrdU antibody (Roche Diagnostics; catalogue number 11647229001) coupled with horseradish peroxidase, the component of commercially available the Cell Proliferation ELISA, BrdU Kit (Roche Diagnostics; catalogue number 11647229001), were subsequently added and detected using TMB (tetramethylbenzidine) substrate. Finally, 1 M sulphuric acid was added to stop the enzymatic reaction. The final product of the reaction was quantified spectrophotometrically at 450 nm using the Infinite M200 Pro microplate reader (Tecan).

### Isobolographic analysis

To classify the types of pharmacological interactions between EX527 and PAX in five various BC cell lines, the type I isobolographic analysis for both parallel and non-parallel concentration-response effects was used. To perform the isobolographic analysis, the anti-viability effects after EX527 and PAX administration in the analysed BC cell lines (MCF7, T47D, MDA-MB-231, MDA-MB-468, BT-549) measured by the *in vitro* MTT assay were transformed to probits. Subsequently, the IC_50_ values for EX527 and PAX (when administered alone and in combination at the fixed ratio of 1:1) were computed from the respective linear equations. To isobolographically analyse the experimental data, the test for parallelism of concentration-response effects for EX527 and PAX (when administered alone) based on the log-probit analysis was used^29^. Subsequently, the experimentally-derived IC_50 mix_ values for the combinations of EX527:PAX (at the fixed ratio of 1:1) were statistically compared with their respective, theoretically calculated IC_50 add_ values (assumed to be additive) with the unpaired Student’s t-test with Welch correction. Details concerning the isobolographic analysis of interactions along with the respective equations for calculating the IC_50 add_ values have been presented earlier^29^^,^^30^.

### Apoptosis analysis

Cytometric analysis was performed to define the percentage of apoptotic cells in reaction to different stimuli. For this purpose, T47D, MCF7, MDA-MB-231, MDA-MB-468 and BT-549 cells at 70% confluence were seeded in 6-well plates (Nunc) at a density of 1 × 10^5^/ml and cultured overnight before incubation with different concentrations of EX527 and PAX used alone or in combination for 48 h. Then, cells were harvested and washed twice with ice-cold PBS. After resuspending, the cells were fixed and permeabilized using the Cytofix/Cytoperm Solution according to the manufacturer’s protocol of PE Active Caspase-3 Apoptosis Kit (BD Pharmingen; San Diego, CA, USA, catalogue number AB_393957). Finally, cells were washed in the Perm/Wash Buffer before intracellular staining with PE-conjugated anti-active caspase-3 monoclonal rabbit antibody (BD Pharmingen; catalogue number AB_393957). Labelled cells were analysed by flow cytometer FACSCalibur^TM^ (Becton Dickinson; San Jose, CA, USA), operating with CellQuest software to quantitatively assess the caspase-3 activity.

### Cell cycle analysis

FACS analysis was performed to define the cell phase distribution after drug treatment. To analyse the effect of the drugs on the cell cycle, T47D, MCF7, MDA-MB-231, MDA-MB-468 and BT-549 cells were seeded at a density of 1 × 10^5^/ml onto 6-well plates (Nunc) and incubated with EX527 and PAX individually or in combination. 48 h after treatments, cells were washed and fixed in 80% cold ethanol at −20 °C for 24h. Then, the cells were resuspended in a solution of propidium iodide (PI) utilising PI/RNase Staining Buffer (BD Biosciences). Cell cycle analysis was performed using the FACSCalibur^™^ flow cytometer (BD Biosciences) equipped with a 488 nm argon-ion laser. Non-commercial flow cytometry analysing software—Cylchred Version 1.0.2 for Windows (University of Wales, Cardiff, Wales, UK) and WinMDI 2.9 for Windows were used. The cells were gated by using a dot plot FL-2 width (x) versus FL-2 area (y)-gate to exclude aggregates and analysed in histograms displaying FL-2 area (yellow-orange fluorescence: 585 nm).

### Zebrafish drug screening assay

For *in vivo* analysis, zebrafish embryos (AB strain) were purchased from the Experimental Medicine Centre (Medical University of Lublin; Lublin, Poland) and were maintained under standard conditions, i.e. 28.5 °C and 14 h light/10 h dark cycle. The zebrafish feeding protocols followed by the Research Animals Department of the Royal Society for the Prevention of Cruelty to Animals (RSPCA). In line with the European Union Directive 2010/63/EU issued on 22 September 2010, zebrafish (Danio rerio) embryos and their earliest life stages are classified as equivalent to *in vitro* cell culture and consequently, they are exempt from regulatory frameworks of animal experiments. Since our study exclusively involved zebrafish larvae younger than 120 hpf (hours post-fertilization) ethical approval was not required.

The doses of drugs utilised for *in vivo* studies were selected based on the survival rates of zebrafish larvae, at predetermined levels of IC_50_, 1/2 IC_50_, 1/4 IC_50_, 1/8 IC_50_, and 1/16 IC_50_ for EX527 and PAX used individually or in combination (mix). The mortality of the larvae that were exposed to the treatment from 2 dpf to 5 dpf was calculated by the number of dead larvae of each sample in triplicate. The zebrafish larvae were cultured in an E3 buffer at a temperature of 32 °C throughout the entire duration of the experiments, up to 120 hpf.

### Zebrafish xenograft injection

T47D, MCF7, MDA-MB-231 and BT-549 BC cells were labelled with Vybrant DiD (Invitrogen; Waltham, MA, USA) dye. For this purpose, the cells at a density of 1 × 10^6^ cells/ml were suspended in a serum-free medium with 5 µL of the cell-labelling solution added per ml. The suspension was incubated for 20 min at 37 °C. Subsequently, the cells were washed twice with PBS, and the pellet resuspended in an appropriate volume of PBS suitable for injection. Next, the stained cells were resuspended in DMEM at the final concentration of 1 × 10^7^ cells/ml. At 48 h post-fertilization (hpf), the zebrafish larvae were manually dechorionated and then subjected to injection. 300 labelled cells were introduced into the larval yolk cavity through an electronically controlled air-pressure microinjection system (Narishige IM-300 Microinjector; Tokyo, Japan). Following injection, the zebrafish larvae with transplanted cells were randomly assigned to the control and drug treatment group (EX527, PAX or EX527:PAX combination), with approximately 30 larvae in each. The zebrafish with the xenografts were maintained in an E3 buffer at a temperature of 32 °C throughout the entire experimental period, up to 120 hpf.

### In vivo zebrafish imaging

Before imaging, zebrafish larvae were anaesthetised using a solution of 0.04 mg/ml ethyl 3-aminobenzoate methanesulfonate tricaine. Imaging of xenografts was conducted at 5 dpf utilising an EVOS M5000 Imaging System (Thermo Fisher Scientific; Waltham) with the Cy5 filter (excitation: 628 nm; emission: 692 nm).

### RNA extraction and quantitative analysis

Total RNA was extracted from 30 xenografted larvae in each studied group using the ExtractMe Total RNA kit (Blirt; Gdansk, Poland). For this purpose, fish larvae with xenografts were lysed using the buffers provided in the kit. Total RNA was purified with microcolumns, including a DNase treatment step, and then eluted with RNase-free water. The RNA concentration was determined using NanoQuant Plate and Tecan Infinite M200 Pro (Männedorf, Switzerland) at 260/280 nm. 1 µg of total isolated RNA was reverse transcribed for cDNA synthesis using the High-Capacity cDNA Reverse Transcription Kit (Applied Biosystems, Waltham) supplemented with RNase Inhibitor (Applied Biosystems). Zebrafish *gpdh* (forward 5′-GTGGAGTCTACTGGTGTCTTC-3′, revers 5′-GTGCAGGAGGCATTGCTTACA-3′) was used as a housekeeping gene. The human *GAPDH* gene (forward 5′-CTCTGCTCCTCCTGTTCGAC-3′, revers 5′-GCCCAATACGACCAAATCC-3′) was used to quantify the number of human cells in the xenografted zebrafish larvae^31^^,^^32^. Then, the human *GAPDH* was used for normalisation of the human: *p21* (forward: 5′-AGGTGGACCTGGAGACTCTCAG-3′, revers: 5′-TCCTCTTGGAGAAGATCAGCCG-3′), *BAX* (forward: 5′-TCAGGATGCGTCCACCAAGAAG-3′, revers: 5′-TGTGTCCACGGCGGCAATCATC-3′), *CCND1* (forward: 5′-TCTACACCGACAACTCCATCCG-3′, revers: 5′-TCTGGCATTTTGGAGAGGAAGTG-3′), *AKT1* (forward: 5′-TGGACTACCTGCACTCGGAGAA-3′, revers: 5′-GTGCCGCAAAAGGTCTTCATGG-3′), *MTOR* (forward: 5′-AGCATCGGATGCTTAGGAGTGG-3′, revers: 5′-CAGCCAGTCATCTTTGGAGACC-3′), *TUBB* (forward: 5′-CTGGACCGCATCTCTGTGTACT-3′, revers: 5′-GCCAAAAGGACCTGAGCGAACA-3′), *S1PR1* (forward: 5′-CCTGTGACATCCTCTTCAGAGC-3′, revers: 5′-CACTTGCAGCAGGACATGATCC-3′), *GNAI1* (forward: 5′-AGCACTGAGTGACTACGACCTG-3′, revers: 5′-GGATGTATCTGTAAACCACTTGTTG-3′) genes analysis. All primers used for quantitative real-time polymerase chain reaction (qPCR) were tested for specificity and sensitivity. qPCR expression analysis was then performed with the LightCycler^®^ 480 II instrument (Roche; Basel, Switzerland) in triplicates on 96 well plates using PowerUp SYBR Green Master Mix (Applied Biosystems). Relative mRNA expression was calculated using the ΔCT subtraction and normalised.

### In silico analysis

The structures of protein-ligand complexes were gathered from the RCSB Protein Data Bank (PDB) database (http://www.rcsb.org/pdb/home/home.do). Visualisations of protein-ligand complexes were prepared using PyMOL (https://pymol.org/2/). The protein-protein interaction pathway analysis was performed using the SIGNOR database (http://signor.uniroma2.it/).

### Statistical analysis

In the study, the experimental data were the results of at least three independent repeated experiments and were expressed as mean ± standard deviation (SD). The experimentally derived IC_50_ and IC_50 mix_ values for EX527 and PAX administered alone or in a 1:1 ratio mixture were determined using log-probit analysis. The unpaired Student’s t-test with Welch’s correction was used to statistically compare the experimentally derived IC_50 mix_ values for the mixture of both drugs with their respective theoretical additive IC_50 add_ values. One-way analysis of variance (ANOVA) and Tukey’s post-hoc testing was used to evaluate the difference group between the indicated values using GraphPad Prism 5.0 (GraphPad Software Inc.; California, U.S.A). Results were statistically relevant if *p* < 0.05 (*p < 0.05, **p < 0.01, ***p < 0.001).

## Results

### EX527 inhibits BC cell viability and proliferation

**Figure 2.**
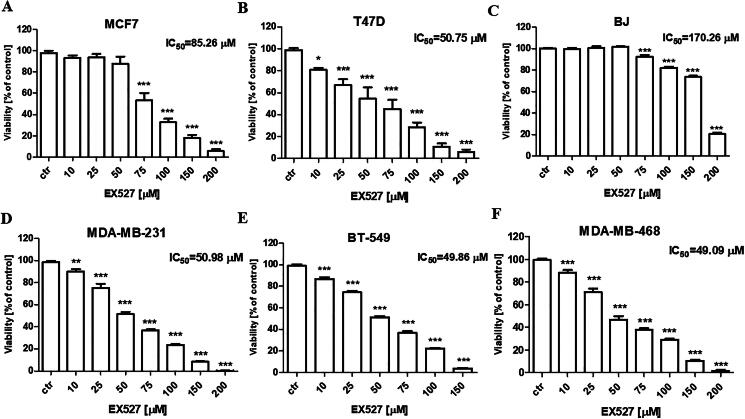
Evaluation of single drug-mediated effects on the viability of the (a) MCF7 and (b) T47D luminal A BC, (c) BJ normal, and (d) MDA-MB-231, (e) BT-549 and (f) MDA-MB-468 BC cells in the MTT assay. BC cells were exposed for 96 h to increasing EX527 concentrations (10–200 µM). The data are presented as the mean ± standard deviation (±SD) of the mean. *p < 0.05, **p < 0.01, ***p < 0.001 by one-way ANOVA, Tukey’s post-hoc test.

Before investigating the combined impact of EX527 and PAX treatment in BC *in vitro* models the influence of EX527 on the cell growth of various BC cell lines, including MCF7 and T47D luminal A, as well as MDA-MB-231, MDA-MB-468 and BT-549 TNBC cells initially were assessed. In the MTT assay EX527 exhibited a dose-dependent inhibition of cell viability in all BC cell lines (Figure 2).

The experimentally derived IC_50_ values for EX527 are presented in Figure 2. We found that EX527 exhibited the cytotoxic effect towards BC cells with IC_50_ values varying from 49.05 ± 8.98 µM to 85.26 ± 9.2 µM, depending on the BC cell line used. More specifically, the experimentally determined log-probit equation allowed for calculating the IC_50_ value for EX527 in the T47D cell line (y = 2.6621x + 1.0863; R^2^ = 0.992) was 50.75 μM, in the MCF7 cell line (y = 4.3531x − 3.4047; R^2^ = 0.961) 85.26 μM, in the MDA-MB-231 cell line (y = 2.2873x + 1.0965; R^2^ = 0.995) was 50.89 μM, in the BT-549 cell line (y = 2.2979x + 1.0988; R^2^ = 0.989) was 49.86 μM and in the MDA-MB-468 cell line (y = 2.8225x + 0.6991; R^2^ = 0.962) 49.04 μM, respectively. The MDA-MB-468 TNBC cells (Figure 2f) were the most sensitive, whereas MCF7 luminal cells (Figure 2a) were the most resistant to the EX527 treatment in the MTT assay. Much weaker activity of EX527 was observed against normal human BJ cell lines, where the IC_50_ was 170.26 μM.

**Figure 3.**
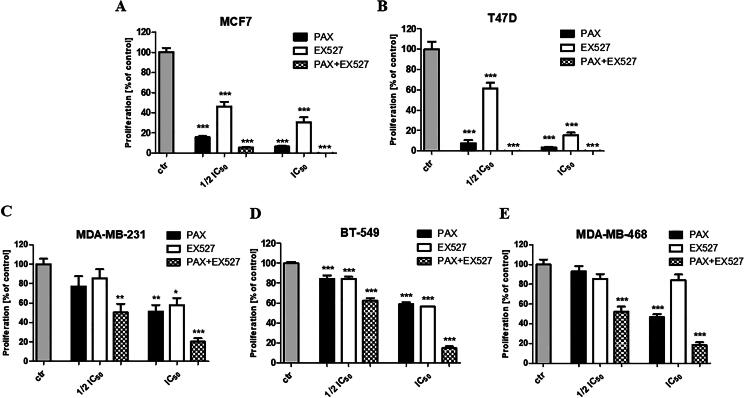
Evaluation of single or combined drug-mediated effects on the proliferation of (a) MCF7, (b) T47D, (c) MDA-MB-231, (d) BT-549, (e) MDA-MB-468 BC cells in the BrdU assay. BC cells were exposed for 48 h to 1/2 and IC_50_ PAX, EX527 or 1:1 PAX:EX527 (1/2 and IC_50 mix)_. The data are presented as the mean ± standard deviation (±SD) of the mean. *p < 0.05, **p < 0.01, ***p < 0.001 by one-way ANOVA, Tukey’s post-hoc test.

In most cases, EX527 administered alone reduced the proliferation of both luminal as well as TNBC cells, as evaluated by BrdU incorporation into cellular DNA in proliferating cells; however, the antiproliferative effect was much more evident in luminal BC cells (Figure 3a,b), than in TNBC (Figure 3c-e).

PAX administered individually inhibited the cell viability of five various (MCF7, T47D, MDA-MB-231, MDA-MB-468 and BT-549) BC cell lines in a concentration-dependent manner. The experimentally determined log-probit equation for PAX in all analysed cell lines, allowing for determining the IC_50_ value, was calculated previously^7^^,^^33^^,^^34^. Interestingly, exposing luminal BC cells to 1/2 or IC_50_ PAX for 48 h in the BrdU assay nearly affected the totality of the cells—the inhibition rate was 15.78 ± 1.219% and 6.483 ± 0.8471% for MCF7 cells, and 7.577 ± 3.078% and 3.688 ± 0.3523 in T47D cells (Figure 3b), respectively. Taken together, all these results remark an effective yet different PAX and EX527 (applied individually) sensitivity between the examined BC cells.

### The combination of EX527 and PAX improves single-treatment effects in BC cells

To address potential cumulative effects in BC models, we subsequently combined effective concentrations of both EX527 and PAX in a constant dilution ratio, and the relative outcome in cell growth was later assessed. T47D, MCF7 luminal and MDA-MB-231, MDA-MB-468 and BT-549 TNBC cells were treated with EX527 and PAX in 1:1 fixed ratio of dose-combinations based on IC_50_ for 96 h. It has been shown that both drugs applied together inhibited the viability of all analysed BC cells in a concentration-dependent fashion, whereas T47D luminal cells were the most resistant and MDA-MB-231 TNBC cells the least sensitive to EX527:PAX co-treatment with the IC_50mix_ = 29.52 ± 3.29 µM and IC_50mix_ = 38.45 ± 5.26 µM, respectively (Table 1).

**Table 1.** The isobolographic analysis of interactions between PAX and EX527 in various tested BC cell lines.

Cell line	IC_50 mix_ (μM)	n _mix_	L-IC_50 add_ (μM)	n _add_	U-IC_50 add_ (μM)	Interaction
MCF7	31.02 ± 7.52	96	22.38 ± 7.44	116	63.18 ± 8.04	Additivity
T47D	29.52 ± 3.29	120	25.38 ± 5.67	188	–	Additivity
MDA-MB-231	38.45 ± 5.26	96	18.40 ± 7.34	164	32.55 ± 8.31	Additivity
BT-549	30.57 ± 3.760	120	24.93 ± 3.60	140	–	Additivity
MDA-MB-468	33.40 ± 3.93	96	24.52 ± 4.49	164	–	Additivity

Results are presented as IC_50_ values (in μM) for the mixtures of PAX and EX527, determined experimentally (IC_50 mix_) and theoretically computed from the equations (L-IC_50 add_ and U-IC_50 add_), which inhibited proliferation in 50% of the tested BC cells. n _mix_: total number of items for the experimental mixture; n _add_: total number of items calculated for the additive mixture of two examined drugs; L-IC_50 add_: lower additive value; U-IC_50 add_: upper additive value.

The effects of EX527 and PAX used in combination on BC cells growth were also monitored using a more sensitive and specific BrdU assay (Figure 3). Significant inhibition of DNA synthesis in response to EX527 and PAX co-treatment has been observed, as evidenced by a decrease in the incorporation of BrdU into DNA. Both concentration combinations (^1^/_2_ and IC_50_) of EX527 and PAX showed decreased proliferation of the studied BC cells in a concentration-dependent manner in relation to single therapy. A significantly stronger antiproliferative activity of EX527:PAX combination was demonstrated in luminal than in TNBC cells. Combination treatment with 1/2 IC_50_ of EX527 and PAX improved single outcomes in both MCF7 and T47D luminal A cell lines. This tendency became even more pronounced at the highest tested doses of the drugs (IC_50_). In contrast to the luminal BC cells, TNBC cells were relatively more resistant to the co-treatment with EX527 and PAX. Collectively, all these data show that the combination of EX527 and PAX impairs BC cell growth more effectively compared with single treatment, suggesting a positive interplay between these two compounds.

### Anti-proliferative concentration–response effects of EX527 and PAX in BC cells

**Figure 4.**
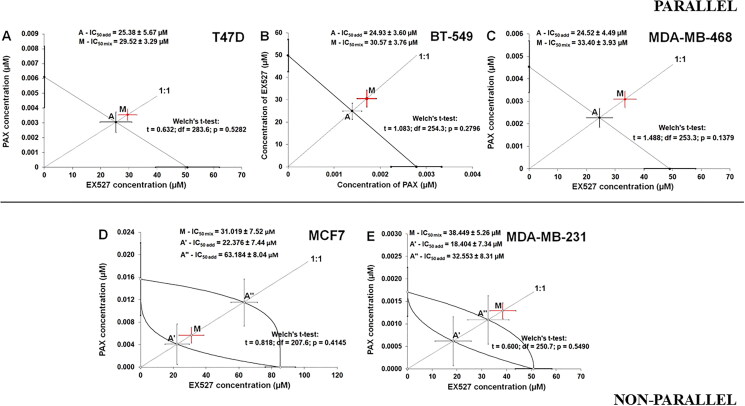
Isobolographic analysis of interaction between PAX and EX527 for parallel (a) T47D, (b) BT-549, (c) MDA-MB-468 and non-parallel (d) MCF7, (e) MDA-MB-231 concentration-response effects in BC cells in the MTT assay. The additive interactions between PAX and EX527 are displayed on isobolograms in T47D, BT-549, MDA-MB-468, MCF7 and MDA-MB-231 BC cells. The dotted diagonal line starting from the point (0,0) corresponds to the mixture of PAX and EX527 at the fixed ratio of 1:1. The points: A (on graphs A-C); A’ and A” (on graphs D-E) depicts the theoretically calculated IC_50 add_ values for the isoboles of additivity for parallel and non-parallel concentration-response effects. The points M represents the IC_50 mix_ values for the total concentrations of the experimental mixture of PAX and EX527 that exerted a 50% anti-viability effect (50% isobole) in the MTT assay. The SEM values are illustrated as horizontal and vertical error bars for every IC_50_ value. The combinations of PAX with EX527 in all analysed BC cell lines were additive in the MTT test because the IC_50 mix_ values for these combinations are placed close to the points A, A’ or A’’. For more detailed information on the isobolographic representation of the data, please refer to^29^.

The test of parallelism between the log-probit concentration–response effects for EX527 and PAX confirmed that the log-probit lines are parallel to one another in the T47D luminal, BT-549 and MDA-MB-468 TNBC cell lines, and non-parallel to each other in the MCF7 luminal and MDA-MB-231 TNBC cell lines (Figure 4a-e).

The mixture of EX527:PAX produced the anti-proliferative effect, whose log-probit equation: y = 1.3282x + 2.7348; R^2^ = 0.9499 (Figure 4a) allowed for calculating the IC_50 mix_ value amounting to 29.52 μM in the T47D cell line. In turn, the combination of EX527 and PAX, at a fixed ratio of 1:1, demonstrated an anti-proliferative effect, and the relationship was modelled using the log-probit equation: y = 2.4131x + 1.4158; R^2^ = 0.9588 (Figure 4b). The equation allowed for the calculation of the IC_50 mix_ value, which amounted to 30.57 μM in the BT-549 cell line. The mixture of EX527 and PAX produced the anti-growth effect, whose log-probit equation: y = 1.8129x + 1.9352; R^2^ = 0.992 allowed for calculating the IC_50 mix_ value = 33.40 μM in the MDA-MB-468 cell line (Figure 4c).

In turn, the mixture of EX527:PAX produced the anti-proliferative effect, whose log-probit equation: y = 1.3686x + 2.9586; R^2^ = 0.899 (Figure 4d) allowed for calculating the IC_50 mix_ value = 31.02 μM in the MCF7 cell line. Finally, the combination of EX527 and PAX (at the fixed ratio of 1:1) produced the anti-proliferative effect with the log-probit equation: y = 2.4277x + 1.1523; R^2^ = 0.958 and the IC_50 mix_ value amounting to 38.45 μM in the MDA-MB-231 cell line (Figure 4e).

### Isobolographic analysis of interaction between EX527 and PAX in BC cells

The type I isobolographic analysis of interactions for mutually collateral concentration-response effects confirmed that the mixture of EX527:PAX exerted an additive interaction in the T47D BC cell line (Figure 4a). The IC_50 mix_ value for this combination in the T47D cell line was 29.52 μM and did not differ significantly from the additively calculated IC_50 add_ value, which was 25.38 μM (t = 0.632; df = 283.6; *p* = 0.528 (Table 1)). The IC_50 mix_ value for this combination in the BT-549 cell line was 30.57 μM and also did not differ significantly from the additively calculated IC_50 add_ value, which was 24.93 μM (Student’s t-test with Welch’s correction: t = 1.083; df = 254.3; *p* = 0.280 (Table 1 and Figure 4b)). Similarly, the mixture of EX527:PAX exerted an additive interaction in the MDA-MB-468 BC cell line (Figure 4c) because the experimentally determined IC_50 mix_ value was 33.40 μM, and did not differ from the additively calculated IC_50 add_ value, which was 24.52 μM (Student’s t-test with Welch’s correction: t = 1.488; df = 253.3; *p* = 0.138 (Table 1)).

The type I isobolographic analysis of interactions for non-parallel concentration-response effects revealed that the mixture of EX527:PAX produced an additive interaction in the MCF7 cell line (Figure 4d) because the experimentally determined IC_50 mix_ value (31.02 μM) did not differ from the additively calculated IC_50 add_ values, which were 22.38 μM (for the lower IC_50 add_) and 63.18 μM (for the upper IC_50 add_), respectively (Student’s t-test with Welch’s correction: t = 0.818; df = 207.6; *p* = 0.415 (Table 1)). Similarly, the mixture of EX527:PAX exerted an additive interaction in the MDA-MB-231 BC cells (Figure 4e). For this combination, the IC_50 mix_ value was 38.45 μM, whereas the additively calculated IC_50 add_ values were 18.40 μM (for the lower IC_50 add_) and 32.55 μM (for the upper IC_50 add_), respectively. No significant difference with the Student’s t-test with Welch’s correction was observed between the IC_50 mix_ and IC_50 add_ values (t = 0.600; df = 250.7; *p* = 0.549 (Table 1)).

### Favourable impact of EX527 and PAX combination on tumour growth inhibition in zebrafish xenografts

**Figure 5.**
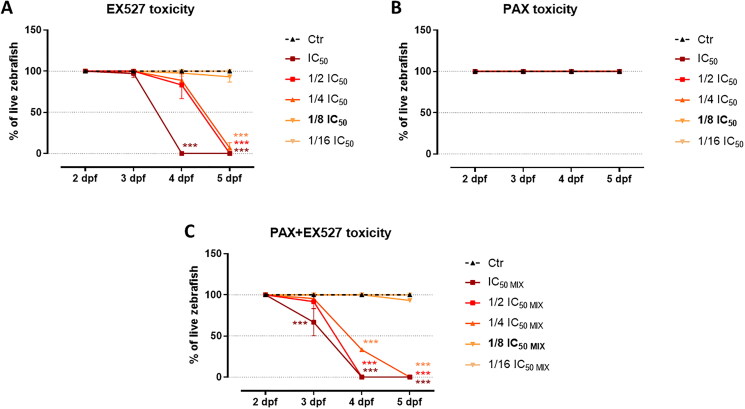
The percentage of zebrafish embryos initiated 2 days post-fertilization (dpf) that survived after 1, 2 or 3 days of treatment with indicated concentrations of (a) EX527, (b) PAX and (c) EX527:PAX (IC_50 mix_). IC_50_ drug doses refer to the effective therapeutic concentrations were determined earlier by the *in vitro* MTT assay. Analyses were performed in triplicates, *n* = 15 per group. Data are presented as mean ± standard deviation of the mean (SD), ***p < 0.001 determined by one-way ANOVA, Tukey’s post-hoc test.

The zebrafish larvae model was used to investigate the anti-tumour potential of EX527 and PAX, applied individually or in combination, in *in vivo* conditions. In the first step, the therapeutic but safe doses of substances were determined experimentally in *in vitro* studies. Initially, it was verified whether these doses and their multiples (1/2, 1/4, 1/8 and 1/16 IC_50_) would induce adverse or side effects in developing zebrafish larvae in comparison to the control group. The drugs were administered to E3 buffer to healthy, tumour-free larvae, and incubated at 32 °C, followed by monitoring for the next 3 consecutive days. It was observed that PAX was well-tolerated by the larvae at each examined concentration (Figure 5b). However, EX527 used individually (Figure 5a) as well as in combination with PAX (Figure 5c) at the three highest concentrations resulted in the death of all larvae within 3 days of treatment. To ensure larval survival and minimise toxicity, the concentration was set at 1/8 of the IC_50_ dose (determined from *in vitro* assays on MDA-MB-231 cells), the highest dose at which nearly all larvae remained viable up to 5 dpf. This concentration allowed for effective experimental observations while maintaining larval viability throughout the study period.

**Figure 6.**
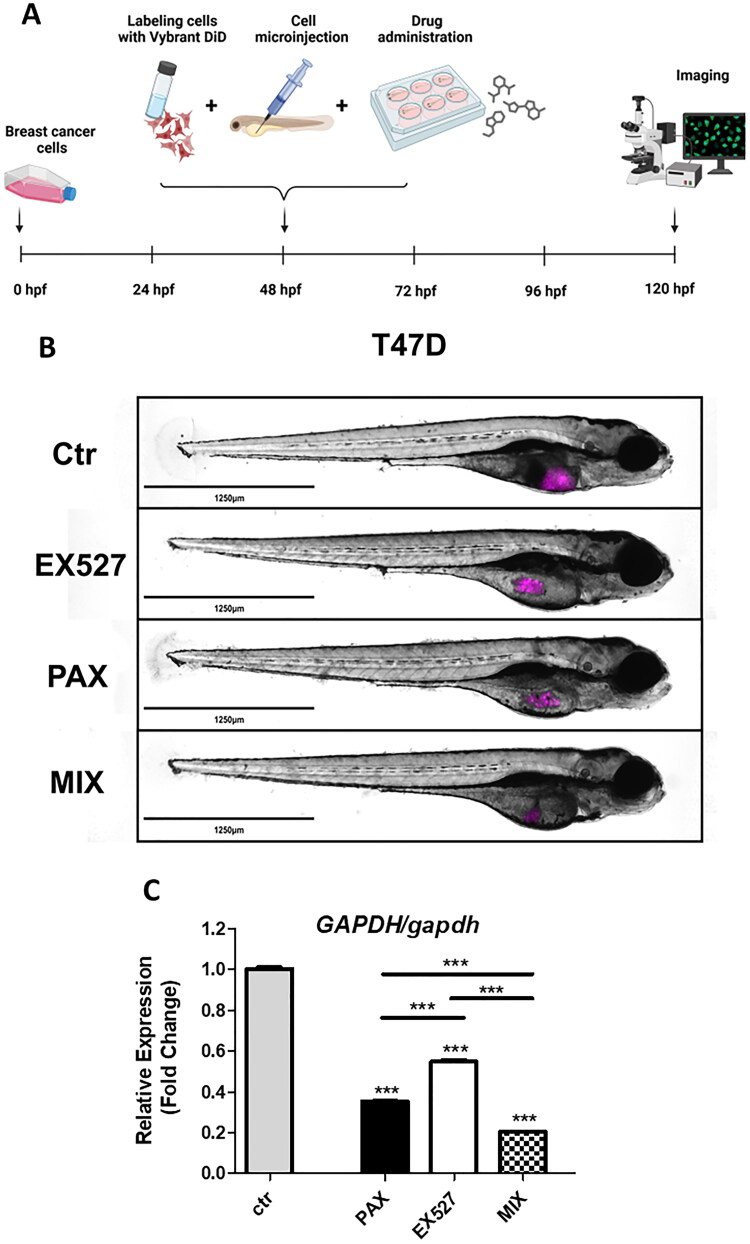
*In vivo* experiments with zebrafish xenografted model. (a) Drug screening scheme with zebrafish larvae model. (b) The impact of 1/8 IC_50_ EX527, PAX and the combination of both compounds (1/8 IC_50 mix_) on T47D xenografted zebrafish model after 3 days of treatment. The representative images of Vybrant^™^ DiD-stained tumour cells after treatment were illustrated. (c) Relative expression of human *GAPDH* versus zebrafish *gapdh* in zebrafish larvae after 1/8 IC_50_ EX527, PAX and EX527:PAX (1/8 IC_50 mix_) treatment. Data are presented as mean ± standard deviation of the mean (SD), ***p < 0.001 determined by one-way ANOVA, Tukey’s post-hoc test.

The antitumor activity of EX527, PAX or EX527:PAX combination was assessed in the zebrafish model of xenotransplantation (Figure 6a). For this purpose, T47D BC cells, stained with Vybrant DiD, were implanted into the yolk of wild-type zebrafish embryos at 2 days dpf, randomly divided into four groups (control group, group treated with 1/8 IC_50_ of EX527, group treated with 1/8 IC_50_ of PAX, and group treated with drug combination (1/8 IC_50 mix_) and treated with 72h with diluted concentrations in E3 buffer. The representative images of Vybrant^™^ DiD-stained tumour cells after treatment were illustrated in Figure 6b. Following the designated time duration, larvae were gathered, and RNA extraction was performed to quantify the ratio of human cells to fish tissue by measuring the human *GAPDH* gene versus the zebrafish *gadph* gene via qRT-PCR method. T47D xenografts treated with both individual compounds and the combination of the drugs exhibited a significant reduction in tumour development compared to the control group. This is reflected in the decrease in fluorescence intensity (Figure 6b) and the reduced relative level of human *GAPDH* versus zebrafish *gapdh* in the examined samples (Figure 6c). The combination of EX527 and PAX was more effective in reducing cancer cells than treatment with individual drugs. All these results indicate that EX527 augments the anti-cancer effect of PAX when used in combination.

### In silico analysis of EX527 and PAX molecular targets pathways

**Figure 7.**
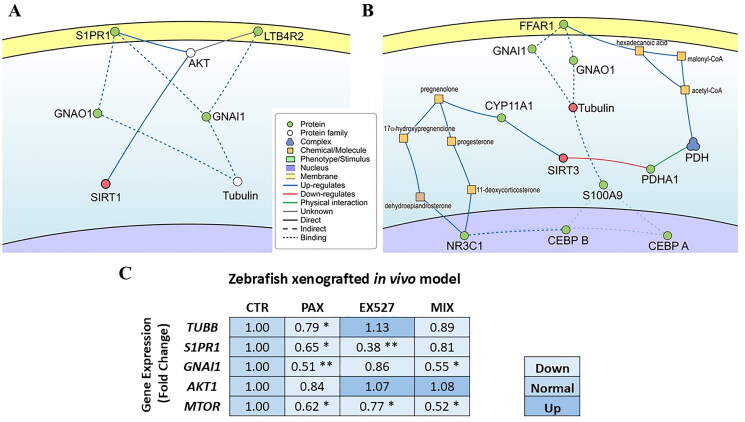
Schematic representation of molecular interaction pathways between (a) SIRT1 or (b) SIRT3 with tubulin, as indicated by SIGNOR. The diagram showcases the proteins and molecules involved in the interaction network between molecular targets of EX527 and PAX. Different shapes and colours denote the nature of the entities, as explained in the legend panel. (c) The effects of PAX, SIRT and PAX:SIRT (1/8 IC_50_ or 1/8 IC_50 mix_) on the gene expressions in zebrafish xenografted BT-549 TNBC *in vivo* model after 72 treatment and analysed by the qRT-PCR. *p < 0.05, **p < 0.01 by one-way ANOVA, Tukey’s post-hoc test.

The protein-protein interaction analysis between SIRT1 and tubulin was conducted using The Signalling Network Open Resource 2.0 (SIGNOR 2.0) (https://doi.org/10.1093/nar/gkv1048). SIGNOR serves as a public repository, storing signalling information in the form of binary causal relationships between biological entities^35^. The assessment for the most direct interaction pathway between the principal molecular targets of EX527 and PAX revealed that SIRT1 and tubulin are interconnected by the shortest pathway consisting of four protein-protein interactions (Figure 7a).

The pathway involves members of the AKT protein family, which includes protein kinase B and kinases Akt-2 and Akt-3. Akt-1 have not been designated, which corresponds to the result obtained in zebrafish BT-549 TNBC xenograft studies, where no significant statistical changes in *AKT1* gene expression were obtained after drug treatment (inhibition of SIRT1) (Figure 7c). The PI3K/AKT/mTOR pathway is a major intracellular network that leads to BC cell proliferation. In the *in vivo* studies, decrease in the *MTOR* expression were detected after PAX, EX527 and PAX/EX527 treatment (1/8 IC_50_) (Figure 7c). In the *in silico* analysis, protein kinase B and kinases Akt-2 and Akt-3 kinases then, through the downregulation of the Leukotriene B4 Receptor 2 (LTB4R2) and up-regulation of the Sphingosine 1-phosphate Receptor 1 (S1PR1), induce a complex effect on levels of the activated Gαi1 and Gαo G-protein subunits, both of which are involved in the tubulin binding. Inhibition of SIRT1 by EX527 and EX527:PAX combination, consistent with *in silico* studies, statistically significantly reduced S1PR1 expression in the zebrafish xenograft model (Figure 7c). Both receptors belong to the superfamily of G protein-coupled receptors (GPCR), transforming extracellular stimuli into intracellular signal by activating members of the G protein family. S1PR1, also known as Endothelial differentiation G-protein coupled receptor 1 (EDG-1) is known to be regulated by Akt by phosphorylation of the T236 residue at the receptor’s intracellular interface^36^. In turn, LTB4R2, alternatively called BLT2, can be phosphorylated by Akt at its T355 residue, located at the intracellular C-terminus, affecting chemotactic responses mediated by this receptor^37^. The α subunits of G proteins, specifically Gi1α, Gsα and Goα, have been demonstrated to enhance the GTPase activity in tubulin, impede the assembly of microtubules, and affect the dynamics of microtubules^38^.

**Figure 8.**
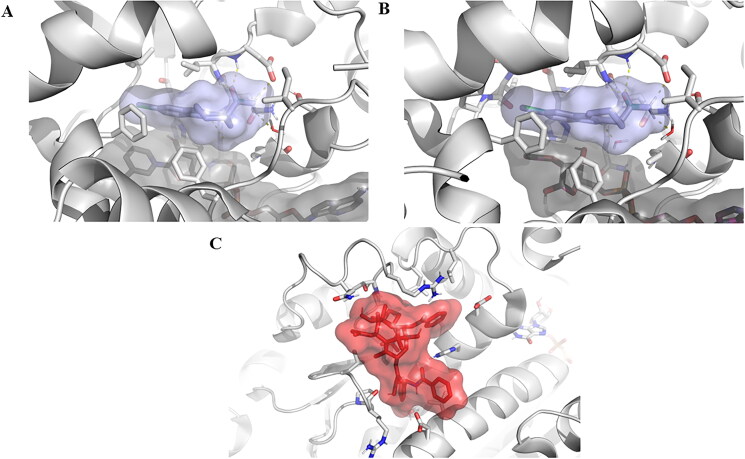
Experimentally solved structures of EX527 analogue bound to (a) SIRT1, as revealed by the PDB entry 4I5I. (b) EX527 bound to SIRT3 in the PDB entry 4BVH, (c) Taxol molecule bound to a tubulin unit, as presented by the PDB entry 6WVR. The distinct chemical nature of the ligands, variations in size, and differences in the characteristics of their respective binding pockets render it improbable for them to engage in competition for the same binding site.

In turn, the most direct interaction pathway between SIRT3 and tubulin unveiled a substantially more intricate network of distant paths requiring seven or more links between nodes (Figure 8b). This network encompasses both up-regulation and down-regulation effects. A summary of all identified pathways is provided in Table 2.

**Table 2.** Molecular pathways between targets of PAX and EX527 identified by SIGNOR.

Pathway name	Pathway string	Distance	Pathway length	Final effect (regulation)
SIRT1→Tubulin	SIRT1**→** AKT**→** S1PR1**→** GNAI1**→** Tubulin	2.505	4	Positive
SIRT1→Tubulin	SIRT1**→** AKT**→** S1PR1**→** GNAO1**→** Tubulin	2.651	4	Positive
SIRT1→Tubulin	SIRT1**→** AKT**―?** LTB4R2**→** GNAI1**→** Tubulin	2.769	4	Unknown
SIRT3→Tubulin	SIRT3**―|** PDHA1**―[]** PDH**→** Acetyl -CoA**→** Hexadecanoic acid**→** FFAR1**→** GNAI1**→** Tubulin	2.685	7	Negative
SIRT3→Tubulin	SIRT3**―|** PDHA1**―[]** PDH**→** Acetyl -CoA**→** Hexadecanoic acid**→** FFAR1**→** GNAO1**→** Tubulin	2.822	7	Negative
SIRT3→Tubulin	SIRT3**―|** PDHA1**―[]** PDH**→** Acetyl-CoA**→** Malonyl-CoA**→** Hexadecanoic acid**→** FFAR1**→** GNAI1**→** Tubulin	2.885	8	Negative
SIRT3→Tubulin	SIRT3**―|** PDHA1**―[]** PDH**→** Acetyl -CoA**→** Malonyl -CoA**→** Hexadecanoic acid**→** FFAR1**→** GNAO1**→** Tubulin	3.022	8	Negative
SIRT3→Tubulin	SIRT3**→** CYP11A1**→** Pregnenolone**→** 17α-Hydroxypregnenolone**→** Dehydroepiandrosterone**→** NR3C1**→** CEBPA**→** S100A9**→** Tubulin	3.727	8	Positive
SIRT3→Tubulin	SIRT3**→** CYP11A1**→** Pregnenolone **→** Progesterone**→** 11-Deoxycorticosterone**→** NR3C1**→** CEBPA**→** S100A9**→** Tubulin	3.727	8	Positive
SIRT3→Tubulin	SIRT3**→** CYP11A1**→** Pregnenolone **→** 17α - Hydroxypregnenolone **→** Dehydroepiandrosterone **→** NR3C1**→** CEBPB**→** S100A9**→** Tubulin	3.753	8	Positive
SIRT3→Tubulin	SIRT3**→** CYP11A1**→** Pregnenolone **→** Progesterone **→** 11-Deoxycorticosterone **→** NR3C1**→** CEBPB**→** S100A9**→** Tubulin	3.753	8	Positive

*Legend for interactions: “→” - up-regulation, “―?” - unknown, “―|” - down-regulation, “―[]” - physical interaction.

In addition to indirect regulatory effects, one could explore the possibility of direct competition between EX527 and PAX for the same binding site. Nevertheless, given the evident differences in ligand structure and binding pocket characteristics (Figure 8), indirect regulatory interactions suggested by SIGNOR appear to be more plausible than a direct competition mechanism.

### Co-administration of EX527 and PAX induces apoptosis in BC cells

**Figure 9.**
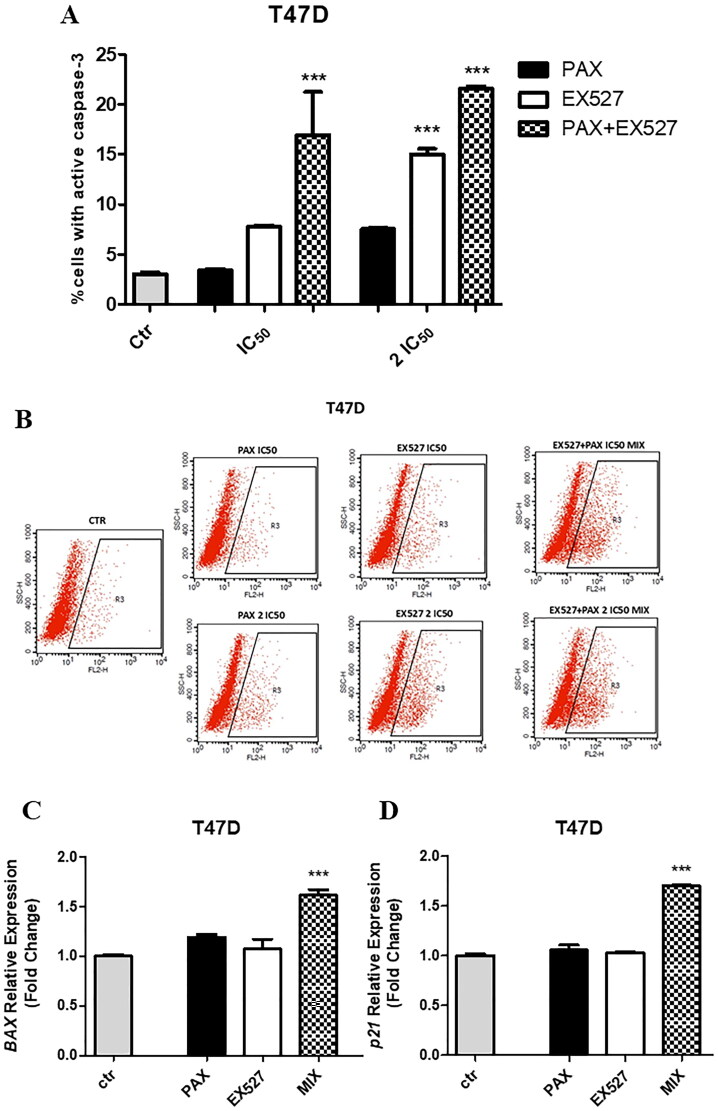
Evaluation of single or combined drug-mediated effects on the induction of apoptosis in T47D BC cells. (a) The % of cells with active caspase-3 determined after PAX and EX527 treatment for 48 h using selected ratios of the IC_50_ determined in the MTT assay. Data are presented as mean ± standard deviation (± SD) of the mean, *** *p* < 0.001 by one-way ANOVA, Tukey’s post-hoc test. (b) Representative dot plots from the FACS analysis of the T47D luminal-type BC cells after a 48h incubation with a medium PAX, EX527 or PAX:EX527 (1:1). R3-apoptotic cells with active caspase-3. (c) *BAX* relative gene expression in zebrafish T47D injected xenograft model after 72 h with 1/8 IC_50_ EX527 or/and PAX treatment determined in qPCR method. *** *p* < 0.001 determined by one-way ANOVA, Tukey’s post-hoc test. (d) *p21* relative expression in zebrafish T47D injected xenograft model after 72 h with 1/8 IC_50_ EX527 or/and PAX treatment determined in qPCR method. *** *p* < 0.001 determined by one-way ANOVA, Tukey’s post-hoc test.

To determine whether the anti-proliferative effect of EX527 in BC cells was associated with apoptosis induction, a population of T47D BC cells with activated caspase-3 by flow cytometry was measured. The obtained results demonstrated that the number of cells with activated caspase-3 significantly and dose-dependently increased in response EX527, PAX and combination in T47D BC cells (Figure 9a,b).

After an exposition of T47D BC cells to IC_50_ PAX, EX527 and combination (IC_50 mix_) the percentage of apoptotic cells was 3.41% ± 0.17, 7.79% ± 0.15, 16.92% ± 4.29, and after 2 IC_50_ − 7.55% ± 0.14, 14.98% ± 0.58, 21.56% ± 0.24, respectively. Additionally, gene expressions connected with apoptosis (*BAX, p21*) were determined in zebrafish T47D injected xenograft model after 72 h with 1/8 IC_50_ EX527 or/and PAX treatment (Figure 9c,d). Although PAX and EX527 used individually did not cause statistically significant differences in *BAX* and *p21* gene expression, their combination showed a marked increase (fold change vs control =1.61 ± 0.10 for *BAX, p* < 0.001 and 1.70 ± 0.01 for *p21, p* < 0.001) in expression of these genes. Summarising, combined exposition of PAX and EX527 augmented the activity of drugs used individually, confirming an additive interaction between these two compounds.

### EX527 and PAX affect cell cycle progression in BC cells

**Figure 10.**
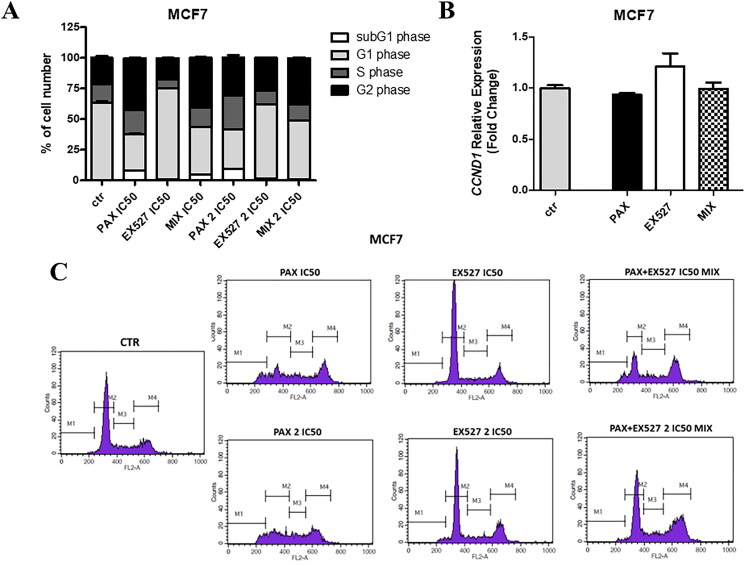
Effect of EX527 or/and PAX on the cell cycle progression in the MCF7 luminal BC cells. (a) BC cells were exposed to individual or concomitant EX527 and PAX treatment for 48 h using selected ratios of the IC_50_ determined in the MTT assay, stained with propidium iodide (PI) and analysed by FACS. The data are presented as the means ± standard deviation (±SD). (b) *CCND1* (cyclin D1) relative gene expression in zebrafish MCF7 injected xenograft model after 72 h with 1/8 IC_50_ EX527 or/and PAX treatment determined in qPCR method. (c) Representative histograms from the FACS analysis of the MCF7 BC cells after a 48h incubation with EX527 or/and PAX. M1-subG1 (pre-G1) phase; M2-G1 phase; M3-S phase; M4-G2 phase.

After finding that the treatment with EX527 and PAX affects BC cell growth, a cell cycle analysis intended to determine the cell phase distribution in reaction to the stimuli was successively performed (Figure 10a,c). Comprehensively, single and combined treatments were performed in MCF7 luminal BC cells for 48 h, and the relative DNA content was later detected by flow cytometry using PI as base pair intercalating dye. The changes in the cell cycle phase distribution were observed in MCF7 cells after both EX527 and PAX treatment (Figure 10a,c). The remarkable subG1- and G2 phase accumulations in reaction to 48h PAX administration were observed. The number of cells in the subG1 phase increased from 0.51 to 8.19% and in the G2 phase from 21.5 to 42.35% after IC_50 PAX_ treatment. Analysis of the subG1 population, which usually includes hypodiploid cells undergoing DNA fragmentation, showed a substantial increase in reaction to PAX (IC_50_ and 2 IC_50_) and EX527:PAX combination (IC_50_) after 48 h compared with untreated cells. Quite the contrary, in MCF7 cells EX527 intensified the G1 cell amount and decreased both S and G2 phases after IC_50_ treatment, while after 2IC_50_, this tendency was not observed (Figure 10a,c). A combination of EX527 and PAX (2 IC_50_) displayed a phase-distribution similar tendency to PAX (Figure 10a,c). Overall, all these findings reveal a different ability in braking cell cycle progression among EX527, PAX and combination. There were no statistically significant changes in *CCND1* (cyclin D1) gene expression after treatment with PAX, EX527 and PAX:EX527 (1/8 IC_50_) in the zebrafish xenograft model with implanted MCF7 cells (Figure 10b).

## Discussion

BC is the most frequently diagnosed tumour worldwide. Despite the increase in the awareness and advancement in diagnosis and screening of BC, treatment possibilities, especially for advanced TNBC—the most aggressive subtype of BC, are still limited^39^. Therefore, new effective therapeutic options for BC are being sought. PAX is a key chemotherapeutic agent in the therapy of BC, but it is burdened with adverse effects, e.g. peripheral neuropathy, haematological toxicities, gastrointestinal disturbances, and hypersensitivity reactions. Thus, the new chemotherapeutics interfering with different molecular mechanisms are still needed^40^. Sirtuins are key regulators for a wide variety of cellular processes such as cancer cell growth, proliferation, differentiation, cell death, or genome instability^41^. So far, no SIRTi has been approved by the Food and Drug Administration (FDA) for cancer therapy^7^. However, several synthetic SIRTi with significant therapeutic activities have been developed. The indole EX527 and its derivatives are the most potent and selective SIRT1 inhibitors. Thus, the therapeutic potential of EX527 has been evaluated in *in vitro* and *in vivo* studies for several diseases, including cancers.

In our studies, we determined for the first time the type of additive pharmacological drug-drug interactions between EX527 and PAX in both luminal and TNBC cell lines with different molecular profiles. We have demonstrated that EX527 has significant cytotoxic and antiproliferative effects in T47D, MCF7 luminal and MDA-MB-231, MDA-MB-468 and BT-549 TNBC cells. Importantly, co-treatment EX527 with PAX was much more effective than single drug treatments. We demonstrated that both agents applied together inhibited the viability of all analysed BC cells in a dose-dependent manner. In contrast to single drug treatment with EX527, T47D luminal cells were the most sensitive, whereas MDA-MB-231 TNBC cells the least sensitive to EX527 and PAX co-treatment. The antiproliferative efficacy of EX527 in a concentration range of 30–100 µM was observed in several different cancer cell lines tested in 2D cell cultures and an approximately three-fold higher concentration range in 3D models *in vitro*. For instance, EX527 reduced viability of pancreatic^42^ and melanoma cells^43^^,^^44^. Additionally, the proliferative and colony formation abilities of glioma cells were inhibited after EX527 treatment. SIRT1 inhibitor exhibited an anticancer effect also on patient-derived glioma cells under 3D culture conditions^27^. Anti-cancer effects of EX527 was also confirmed in cervical cancer cells in a similar concentration range^45^. EX527 suppressed the malignant phenotype, proliferation and colony formation in HeLa cells^28^. Interestingly, EX527 impaired the growth of SiHa cervical cancer cells, but not of immortalised HaCaT non-cancerous cells above 30 μM^28^. EX527 has been extensively studied not only in solid tumours but also in haematological malignancies. It has been reported that SIRT1 inhibitor restrained cell growth with IC_50_ ranging from 50–100 µM in MEC-2 and JVM-3 B-cell chronic lymphocytic leukaemia cell lines^46^.

In our studies for the first time, we assessed the activity of EX527 used individually or in combination with PAX in the zebrafish xenografted *in vivo* model. A significant effect of EX527, PAX or EX527:PAX on the reduction of the tumour size was confirmed. Moreover, at the concentrations where EX527 exhibited efficacy (6 µM) in diminishing tumour size, there was no notable decrease observed in the viability of the zebrafish larvae. Similar study performed on mouse xenograft model demonstrated that SIRT1 inhibition by EX257 enhances the sensitivity of HPV+ head and neck cancer cells to genotoxic agents, including cisplatin^47^, showing that EX257 display similar activity against different cancer types *in vivo*, and not restricted to BC only.

To further analyse the mechanism by which EX527 inhibits the proliferation of BC cells, a cell cycle analysis using FACS were performed. Interestingly, a different cell cycle distribution after EX527 and PAX was observed. Remarkable cell accumulation in subG1- and G2 phases after PAX treatment was observed. In contrast, EX527 increased cells amount in the G1 and decreased in both, S and G2 phases. Similar to our results, literature data indicate that SiHa and Hela cervical cancer cells treated with EX527 displayed growth arrest in G1/subG1 phase of cell cycle^28^. Additionally, western blot analysis demonstrated that the protein amount of CDK4 or CDK6 and cyclin D1 were decreased after EX527 treatment in Hela cells^28^ which effect we did not observe at mRNA level in MCF7 BC cells. In our study, a combined treatment with EX527 and PAX (IC_50_) displayed a phase distribution similar to PAX—the increase in the cell amount in G2 phase and a decrease in S phase, suggesting that cell cycle perturbations were attributed rather to PAX, than EX257 treatment.

In contrast, the pro-apoptotic (the number of cells with activated caspase-3) effect of EX527 and PAX used individually or in combination increased in a dose-dependent manner in BC cells tested. Interestingly, *p21* and *BAX* gene expressions were augmented after PAX:EX527 treatment compared to the individual drug applications, giving a hint of potential mechanism of combined drug action in tested BC cells. Similarly, other reports demonstrated that EX527 induced apoptosis in Huh7 and HepG2 hepatocellular carcinoma cells, with no changes in the stimulation of necrotic death as assessed by determination of the Annexin V-/PI+ ratio^44^. Treatment with EX527 also increased the number of apoptotic cells in glioma, correlated with increased p21, p53 and acetylated p53, as well asB-cell lymphoma 2 (Bcl-2)-associated-X-protein with a simultaneous reduction of Bcl-2 proteins expression^27^. The different mechanism of apoptosis activation was reported in both MEC-2 and JVM-3 primary CLL cell lines following incubation with EX527 (100 µM), where a significant increase in mitochondrial superoxide was detected^46^, suggesting ROS-dependent mitochondrial pathway. On the opposite, increase in number of apoptotic cells were not markedly observed after EX527 treatment in HeLa cells. Consistent with this observation, PARP and caspase-3 were not cleaved in cells treated with EX527 compared with the control. Additionally, LC3B and beclin-1—key autophagy proteins, were not activated after incubation with EX527^28^. These data could indicate cell-dependent induction of apoptosis after EX527 treatment.

With the use of the advanced isobolography method, we have determined additive pharmacological drug-drug interaction between EX527 and PAX in T47D, MCF7, MDA-MB-231, MDA-MB-468 and BT-549 BC cells, proving that these compounds can be used in BC combined therapy in the future. Additionally, the *in silico* data suggest the possibility of an indirect mode of interaction between EX527 and PAX, occurring through a network of intracellular interactions, and the prevalence of a particular path can affect the eventual outcome of simultaneous administration. Analysis of the protein-protein interaction network with SIGNOR has shown the possibility of interactions between drugs targeting those targets through SIRT1-AKT-S1PR1-GNAI1/GNAO1-Tubulin pathway, which positively corresponds with the data obtained from the gene analysis in the zebrafish xenografted model. In the context of the presented findings, the SIGNOR analysis on protein-protein interactions provides valuable insight into the molecular intricacies underpinning the effects of EX527 in combination with PAX. The protein-protein interaction pathways connecting molecular targets of both ligands were found, and the putative crucial nodes of the protein-protein interaction pathways were identified. Harnessing this knowledge can pave the way for optimising therapeutic combinations, potentially unlocking more effective and tailored treatment regimens for BC patients.

Similarly to our research, EX527 has been extensively studied in combination with other active agents in numerous types of cancer, showing their beneficial effects. EX527 significantly suppressed the proliferation and cisplatin resistance in endometrial carcinoma cell lines. On the other hand, SIRT1 overexpression induced tumour growth and cisplatin resistance in nude mice^48^. Similarly, the chemosensitivity of H460-R lung cancer cells to cisplatin was increased after EX527 treatment (IC_50_ was decreased nearly twice—from 11.4 to 6.5 μg/ml). Induction of apoptosis and DNA damage were observed after cisplatin treatment in the presence of SIRT1 inhibition by EX527. The half-life of XRCC1 protein was shortened significantly in the presence of EX527, indicating that SIRT1 inhibits XRCC1 protein degradation^49^. In another study, EX527 enhanced the sensitivity of PANC-1 pancreatic cancer cells to gemcitabine through the induction of apoptosis. However unexpectedly, EX527 promoted PANC-1 xenograft tumour growth in SCID mice compared to the control group. Combination of EX527 and gemcitabine displays no synergistic effect compared to gemcitabine alone *in vivo*^42^. Comparing to our results, these studies demonstrated that combination therapy with EX257 and other compounds is dependent on the type of drug used and cancer cells origin. On the other hand, the ineffective drug treatment could be changed by SIRT1 inhibition. The addition of EX527 significantly increased sorafenib cytotoxicity in 2D cultures in hepatocellular carcinoma cell lines. Interestingly, although sorafenib used individually did not affect cellular migration and invasion, combined treatment reduced the migration in 2D cultures. EX527 used in a combination treatment significantly increased sorafenib cytotoxicity also in 3D spheroids cultures created from and HepG2 cells reducing proliferation and increasing apoptosis of spheroid core regions compared to sorafenib single treatment as quantified by PCNA, Ki-67 and cyclin D1 staining^50^. Thus, combining the EX257 with other drugs may constitute an interesting approach for treatment of different cancer types.

## Conclusions

The combined exposition of BC to PAX and EX527 augmented the activity of drugs used individually in both *in vitro* and *in vivo* BC models, confirming an additive interaction between these two compounds, and making this drug combination a subject of clinical interest as a promising approach in the future BC regimens. However, additional studies are needed to elucidate the targets and molecular mechanisms of EX527:PAX activity in other pre-clinical, e.g. mammalian models.

## Data Availability

The datasets presented in the current study are available from the corresponding author upon reasonable request.

## References

[1] Arnold M, Morgan E, Rumgay H, Mafra A, Singh D, Laversanne M, Vignat J, Gralow JR, Cardoso F, Siesling S, et al. Current and future burden of breast cancer: global statistics for 2020 and 2040. Breast. 2022;66:15–23. 36084384 10.1016/j.breast.2022.08.010 PMC9465273

[2] Gupta SR. Prediction time of breast cancer tumor recurrence using machine learning. Cancer Treat Res Commun. 2022;32:100602. 35797887 10.1016/j.ctarc.2022.100602

[3] Rakha EA, Green AR. Molecular classification of breast cancer: what the pathologist needs to know. Pathology. 2017;49(2):111–119. 28040199 10.1016/j.pathol.2016.10.012

[4] Eliyatkin N, Yalcin E, Zengel B, Aktaş S, Vardar E. Molecular classification of breast carcinoma: from traditional, old-fashioned way to a new age, and a new way. J Breasth Health. 2015;11(2):59–66. 10.5152/tjbh.2015.1669 PMC5351488 28331693

[5] Viale G. The current state of breast cancer classification. Ann Oncol. 2012;23 Suppl 10(10):x207–210. 22987963 10.1093/annonc/mds326

[6] Massafra R, Comes MC, Bove S, Didonna V, Diotaiuti S, Giotta F, Latorre A, La Forgia D, Nardone A, Pomarico D, et al. A machine learning ensemble approach for 5- and 10-year breast cancer invasive disease event classification. PLoS One. 2022;17(9):e0274691. 36121822 10.1371/journal.pone.0274691 PMC9484691

[7] Wawruszak A, Okon E, Telejko I, Czerwonka A, Luszczki J. Additive pharmacological interaction between sirtuin inhibitor cambinol and paclitaxel in MCF7 luminal and MDA-MB-231 triple-negative breast cancer cells. Pharmacol Rep. 2022;74(5):1011–1024. 35900723 10.1007/s43440-022-00393-w PMC9585000

[8] Dworkin AM, Huang THM, Toland AE. Epigenetic alterations in the breast: implications for breast cancer detection, prognosis and treatment. Semin Cancer Biol. 2009;19(3):165–171. 19429480 10.1016/j.semcancer.2009.02.007 PMC2734184

[9] Nair SS, Kumar R. Chromatin remodeling in cancer: a gateway to regulate gene transcription. Mol Oncol. 2012;6(6):611–619. 23127546 10.1016/j.molonc.2012.09.005 PMC3538127

[10] Chojdak-Łukasiewicz J, Bizoń A, Waliszewska-Prosół M, Piwowar A, Budrewicz S, Pokryszko-Dragan A. Role of sirtuins in physiology and diseases of the central nervous system. Biomedicines. 2022;10(10):2434. 36289696 10.3390/biomedicines10102434 PMC9598817

[11] Yu L, Li Y, Song S, Zhang Y, Wang Y, Wang H, Yang Z, Wang Y. The dual role of sirtuins in cancer: biological functions and implications. Front Oncol. 2024;14:1384928. 38947884 10.3389/fonc.2024.1384928 PMC11211395

[12] Jiang Y, Liu J, Chen D, Yan L, Zheng W. Sirtuin inhibition: strategies, inhibitors, and therapeutic potential. Trends Pharmacol Sci. 2017;38(5):459–472. 28389129 10.1016/j.tips.2017.01.009

[13] Xu X, Zhang Q, Wang X, Jin J, Wu C, Feng L, Yang X, Zhao M, Chen Y, Lu S, et al. Discovery of a potent and highly selective inhibitor of SIRT6 against pancreatic cancer metastasis in vivo. Acta Pharm Sin B. 2024;14(3):1302–1316. 38487000 10.1016/j.apsb.2023.11.014 PMC10935062

[14] Rifaï K, Idrissou M, Penault-Llorca F, Bignon YJ, Bernard-Gallon D. Breaking down the contradictory roles of histone deacetylase sirt1 in human breast cancer. Cancers (Basel).)2018;10(11):409. 30380732 10.3390/cancers10110409 PMC6266715

[15] Scarano N, Brullo C, Musumeci F, Millo E, Bruzzone S, Schenone S, Cichero E. Recent advances in the discovery of sirt1/2 inhibitors via computational methods: a perspective. Pharmaceuticals. 2024;17(5):601. 38794171 10.3390/ph17050601 PMC11123952

[16] Krushkal J, Zhao Y, Roney K, Zhu W, Brooks A, Wilsker D, Parchment RE, McShane LM, Doroshow JH. Association of changes in expression of HDAC and SIRT genes after drug treatment with cancer cell line sensitivity to kinase inhibitors. Epigenetics. 2024;19(1):2309824. 38369747 10.1080/15592294.2024.2309824 PMC10878021

[17] Chang N, Li J, Lin S, Zhang J, Zeng W, Ma G, Wang Y. Emerging roles of SIRT1 activator, SRT2104, in disease treatment. Sci Rep. 2024;14(1):5521. 38448466 10.1038/s41598-024-55923-8 PMC10917792

[18] Giri S. Abstract 3238: therapeutic targeting of the genome guardian sirtuins in metastatic prostate cancer. Cancer Res. 2024;84(6_Supplement):3238–3238.

[19] Fu X-T, Qie J-B, Chen J-F, Gao Z, Li X-G, Feng S-R, Dong E-F, Shi Y-H, Tang Z, Liu W-R, et al. Inhibition of SIRT1 relieves hepatocarcinogenesis via alleviating autophagy and inflammation. Int J Biol Macromol. 2024;278(Pt 1):134120. 39074701 10.1016/j.ijbiomac.2024.134120

[20] de Castro LR, de Oliveira LD, Milan TM, Eskenazi APE, Bighetti-Trevisan RL, de Almeida OGG, Amorim MLM, Squarize CH, Castilho RM, de Almeida LO, et al. Up-regulation of TNF-alpha/NFkB/SIRT1 axis drives aggressiveness and cancer stem cells accumulation in chemoresistant oral squamous cell carcinoma. J Cell Physiol. 2024;239(2):e31164. 38149816 10.1002/jcp.31164

[21] Haggagy MG, Ahmed LA, Sharaky M, Elhefnawi MM, Omran MM. SIRT1 as a potential key regulator for mediating apoptosis in oropharyngeal cancer using cyclophosphamide and all-trans retinoic acid. Sci Rep. 2024;14(1):41. 38167952 10.1038/s41598-023-50478-6 PMC10761886

[22] Zhang X, Dong Y, Li W, He M, Shi Y, Han S, Li L, Zhao J, Li L, Huo J, et al. The mechanism by which SIRT1 regulates autophagy and EMT in drug-resistant oesophageal cancer cells. Life Sci. 2024;343:122530. 38401628 10.1016/j.lfs.2024.122530

[23] Yang X, Li S, Xu C, Liu S, Zhang X, Lian B, Li M. Sirtuin1 (sirt1) regulates the glycolysis pathway and decreases cisplatin chemotherapeutic sensitivity to esophageal squamous cell carcinoma. Cancer Biol Ther. 2024;25(1):2365449. 38865161 10.1080/15384047.2024.2365449 PMC11174053

[24] Kang JA, Kim YJ, Jang KY, Moon HW, Lee H, Lee S, Song HK, Cho SW, Yoo YS, Han HG, et al. SIRT1 ISGylation accelerates tumor progression by unleashing SIRT1 from the inactive state to promote its deacetylase activity. Exp Mol Med. 2024;56(3):656–673. 38443596 10.1038/s12276-024-01194-2 PMC10985095

[25] Broussy S, Laaroussi H, Vidal M. Biochemical mechanism and biological effects of the inhibition of silent information regulator 1 (SIRT1) by EX-527 (SEN0014196 or selisistat). J Enzyme Inhib Med Chem. 2020;35(1):1124–1136. 32366137 10.1080/14756366.2020.1758691 PMC7241506

[26] Fajardo-Orduña GR, Ledesma-Martínez E, Aguiñiga-Sanchez I, Weiss-Steider B, Santiago-Osorio E. Role of SIRT1 in chemoresistant leukemia. Int J Mol Sci. 2023;24(19):14470. 37833921 10.3390/ijms241914470 PMC10573076

[27] Wang T, Li X, Sun SL. EX527, a Sirt-1 inhibitor, induces apoptosis in glioma via activating the p53 signaling pathway. Anticancer Drugs. 2020;31(1):19–26. 31490284 10.1097/CAD.0000000000000824

[28] Kim HW, Kim SA, Ahn SG. Sirtuin inhibitors, EX527 and AGK2, suppress cell migration by inhibiting HSF1 protein stability. Oncol Rep. 2016;35(1):235–242. 26530275 10.3892/or.2015.4381

[29] Luszczki JJ. Isobolographic analysis of interaction between drugs with nonparallel dose-response relationship curves: a practical application. Naunyn Schmiedebergs Arch Pharmacol. 2007;375(2):105–114. 17333129 10.1007/s00210-007-0144-z

[30] Wawruszak A, Luszczki JJ, Grabarska A, Gumbarewicz E, Dmoszynska-Graniczka M, Polberg K, Stepulak A. Assessment of interactions between cisplatin and two histone deacetylase inhibitors in MCF7, T47D and MDA-MB-231 human breast cancer cell lines—an isobolographic analysis. PLoS One. 2015;10(11):e0143013. 26580554 10.1371/journal.pone.0143013 PMC4651465

[31] Al-Samadi A, Tuomainen K, Kivimäki A, Salem A, Al-Kubati S, Hyytiäinen A, Parikka M, Mesimäki K, Wilkman T, Mäkitie A, et al. PCR-based zebrafish model for personalised medicine in head and neck cancer. J Transl Med. 2019;17(1):235. 31331338 10.1186/s12967-019-1985-1 PMC6647158

[32] Avci ME, Keskus AG, Targen S, Isilak ME, Ozturk M, Atalay RC, Adams MM, Konu O. Development of a novel zebrafish xenograft model in ache mutants using liver cancer cell lines. Sci Rep. 2018;8(1):1570. 29371671 10.1038/s41598-018-19817-w PMC5785479

[33] Wawruszak A, Luszczki J, Czerwonka A, Okon E, Stepulak A. Assessment of pharmacological interactions between SIRT2 inhibitor AGK2 and paclitaxel in different molecular subtypes of breast cancer cells. Cells. 2022;11(7):1211. 35406775 10.3390/cells11071211 PMC8998062

[34] Wawruszak A, Luszczki J, Okon E, Czerwonka A, Stepulak A. Antagonistic pharmacological interaction between sirtuin inhibitor cambinol and paclitaxel in triple-negative breast cancer cell lines: an isobolographic analysis. Int J Mol Sci. 2022;23(12):6458. 35742901 10.3390/ijms23126458 PMC9223454

[35] Licata L, Lo Surdo P, Iannuccelli M, Palma A, Micarelli E, Perfetto L, Peluso D, Calderone A, Castagnoli L, Cesareni G, et al. SIGNOR 2.0, the SIGnaling network open resource 2.0: 2019 update. Nucleic Acids Res. 2020;48(D1):D504–D510. 31665520 10.1093/nar/gkz949 PMC7145695

[36] Lee MJ, Thangada S, Paik JH, Sapkota GP, Ancellin N, Chae SS, Wu M, Morales-Ruiz M, Sessa WC, Alessi DR, et al. Akt-mediated phosphorylation of the G protein-coupled receptor EDG-1 is required for endothelial cell chemotaxis. Mol Cell. 2001;8(3):693–704. 11583630 10.1016/s1097-2765(01)00324-0

[37] Wei JD, Kim JY, Kim JH. BLT2 phosphorylation at Thr355 by Akt is necessary for BLT2-mediated chemotaxis. FEBS Lett. 2011;585(22):3501–3506. 22044535 10.1016/j.febslet.2011.10.037

[38] Roychowdhury S, Panda D, Wilson L, Rasenick MM. G protein α subunits activate tubulin GTPase and modulate microtubule polymerization dynamics. J Biol Chem. 1999;274(19):13485–13490. 10224115 10.1074/jbc.274.19.13485

[39] Akram M, Iqbal M, Daniyal M, Khan AU. Awareness and current knowledge of breast cancer. Biol Res. 2017;50(1):33. 28969709 10.1186/s40659-017-0140-9 PMC5625777

[40] Shenoy PK, Ebrahim I, Skaria, AM, AM. Toxicity profile of weekly regimen of paclitaxel in patients with non-metastatic breast cancer-a real world experience (India). Onkol i Radioter. 2021;15(2):1–4.

[41] Bosch-Presegué L, Vaquero A. The dual role of sirtuins in cancer. Genes Cancer. 2011;2(6):648–662. 21941620 10.1177/1947601911417862 PMC3174263

[42] Oon CE, Strell C, Yeong KY, Östman A, Prakash J. SIRT1 inhibition in pancreatic cancer models: contrasting effects in vitro and in vivo. Eur J Pharmacol. 2015;757:59–67. 25843411 10.1016/j.ejphar.2015.03.064

[43] Wilking MJ, Singh CK, Nihal M, Ndiaye MA, Ahmad N. Sirtuin deacetylases: a new target for melanoma management. Cell Cycle. 2014;13(18):2821–2826. 25486469 10.4161/15384101.2014.949085 PMC4614878

[44] Ceballos MP, Decándido G, Quiroga AD, Comanzo CG, Livore VI, Lorenzetti F, Lambertucci F, Chazarreta-Cifre L, Banchio C, Alvarez MdL, et al. Inhibition of sirtuins 1 and 2 impairs cell survival and migration and modulates the expression of P-glycoprotein and MRP3 in hepatocellular carcinoma cell lines. Toxicol Lett. 2018;289:63–74. 29545174 10.1016/j.toxlet.2018.03.011

[45] Wössner N, Alhalabi Z, González J, Swyter S, Gan J, Schmidtkunz K, Zhang L, Vaquero A, Ovaa H, Einsle O, et al. Sirtuin 1 inhibiting thiocyanates (S1th)—A new class of isotype selective inhibitors of NAD+ dependent lysine deacetylases. Front Oncol. 2020;10:657. 32426286 10.3389/fonc.2020.00657 PMC7203344

[46] Bhalla S, Gordon LI. Functional characterization of NAD dependent de-acetylases SIRT1 and SIRT2 in B-cell chronic lymphocytic leukemia (CLL). Cancer Biol Ther. 2016;17(3):300–309. 26794150 10.1080/15384047.2016.1139246 PMC4847985

[47] Lo Cigno I, Calati F, Girone C, Borgogna C, Venuti A, Boldorini R, Gariglio M. SIRT1 is an actionable target to restore p53 function in HPV-associated cancer therapy. Br J Cancer. 2023;129(11):1863–1874. 37838812 10.1038/s41416-023-02465-x PMC10667542

[48] Asaka R, Miyamoto T, Yamada Y, Ando H, Mvunta DH, Kobara H, Shiozawa T. Sirtuin 1 promotes the growth and cisplatin resistance of endometrial carcinoma cells: a novel therapeutic target. Lab Invest. 2015;95(12):1363–1373. 26367491 10.1038/labinvest.2015.119

[49] Yousafzai NA, Zhou Q, Xu W, Shi Q, Xu J, Feng L, Chen H, Shin VY, Jin H, Wang X, et al. SIRT1 deacetylated and stabilized XRCC1 to promote chemoresistance in lung cancer. Cell Death Dis. 2019;10(5):363. 31043584 10.1038/s41419-019-1592-3 PMC6494911

[50] Ceballos MP, Angel A, Delprato CB, Livore VI, Ferretti AC, Lucci A, Comanzo CG, Alvarez MdL, Quiroga AD, Mottino AD, et al. Sirtuin 1 and 2 inhibitors enhance the inhibitory effect of sorafenib in hepatocellular carcinoma cells. Eur J Pharmacol. 2021;892:173736. 33220273 10.1016/j.ejphar.2020.173736

